# Protective Effect of Boric Acid Against Ochratoxin A-Induced Toxic Effects in Human Embryonal Kidney Cells (HEK293): A Study on Cytotoxic, Genotoxic, Oxidative, and Apoptotic Effects

**DOI:** 10.1007/s12011-024-04194-5

**Published:** 2024-05-07

**Authors:** Aşkın Tekin, Adem Güner, Tamer Akkan

**Affiliations:** 1 Faculty of Health Sciences, Department of Occupational Health and Safety,, Sinop University, Sinop, Türkiye; 2 Şebinkarahisar Vocational School of Health Services, Giresun,, Giresun University, Giresun, Türkiye; 3 Faculty of Arts and Science, Biology Department of Biology, Giresun University, Giresun, Türkiye

**Keywords:** Ochratoxin A, Boric acid, Protective effect, Genotoxicity

## Abstract

The present study evaluates the protective properties of boric acid (BA) against the toxic effects induced by ochratoxin A (OTA) in human embryonic kidney cells (HEK293). The focus is on various parameters such as cytotoxicity, genotoxicity, oxidative stress, and apoptosis. OTA is a known mycotoxin that has harmful effects on the liver, kidneys, brain, and nervous system. BA, on the other hand, a boron-based compound, is known for its potential as a vital micronutrient with important cellular functions. The results show that BA administration not only increases cell viability but also mitigates the cytotoxic effects of OTA. This is evidenced by a reduction in the release of lactate dehydrogenase (LDH), indicating less damage to cell membranes. In addition, BA shows efficacy in reducing genotoxic effects, as the frequency of micronucleus (MN) and chromosomal aberrations (CA) decreases significantly, suggesting a protective role against DNA damage. In addition, the study shows that treatment with BA leads to a decrease in oxidative stress markers, highlighting its potential as a therapeutic intervention against the deleterious effects of OTA. These results emphasize the need for further research into the protective mechanisms of boron, particularly BA, in combating cell damage caused by OTA.

## Introduction

Mycotoxins are secondary metabolites produced by the fungi such as *Aspergillus*, *Alternaria*, *Claviceps*, *Fusarium*, *Penicillium*, and *Stachybotrys* [1]. More than 300 mycotoxins have been identified, but the most important toxicologically are aflatoxins, zearalenone, trichothecenes, fumonisins, and ochratoxins, as they have carcinogenic and immunosuppressive properties and can also inhibit human and animal growth and thus impair productivity [2]. Ochratoxin A (OTA) which is produced by several *Aspergillus* species and *Penicillium verrucosum* structurally has a para-chlorophenolic group containing a dihydroisocoumarin moiety that is amide-linked to L-phenylalanine. Its chemical name is L-phenylalanine-N-[(5-chloro-3,4-dihydro-8-hydroxy-3-methyl-1-oxo-1H-2-benzopyrane-7-yl)carbonyl]-(R)-isocoumarin [3]. OTA can contaminate cereal grains, cereal products, nuts, dried fruits, dried meats, blood sausages, spices, meat, milk, wine, beer, coffee, infant formula, and baby foods [4]. OTA has several toxicological effects such as teratogenic, hepatotoxic, nephrotoxic, carcinogenic, and immunosuppressive. The kidney has been recognized as the primary target organ for OTA, which has been demonstrated in almost all animal species [5, 6].

OTA is known to be a potent nephrotoxin because it accumulates in the epithelial cells of the proximal tubule in the kidney. The mode of action of OTA is not yet clearly understood and seems to be very complex. Inhibition of protein synthesis, oxidative stress/nitrosative stress, DNA damage/genotoxicity, apoptosis/pyroptosis, lipotoxicity, cell cycle arrest, mitochondrial dysfunction/ATP, miRNA, autophagy, EMT/fibrosis/tight junction, and inflammatory responses are possibly involved in its toxic action.

The mechanism of action of OTA is still unclear. Therefore, it is difficult to develop effective strategies to alleviate the toxicity caused by OTA at the cellular level. Various natural compounds and drugs such as lycopene [7], a-tocopherol [8], catechins [9], resveratrol [10], N-acetylcysteine [11], L-Dopa [12], phenylalanine [13], and aspartame [14] have certain antagonistic effects to the renal toxicity of OTA.

Boron and its compounds have long been known for their positive effect on the metabolism of humans and animals. Boron is not known to be indispensable for humans, but is instead considered by the WHO to be a probably essential element [15]. Boron has outstanding effects on human health, including its influence on bone development and regeneration, wound healing, the production and metabolism of sex steroids and vitamin D, and the absorption and utilization of calcium and magnesium. In addition, boron has anti-inflammatory effects that can help alleviate arthritis and improve brain function, and it has shown such significant anticancer effects that boron-containing compounds are now used in the treatment of various types of cancer [16].

Boric acid (BA) and the sodium borate are commonly used as an antiseptic, bactericide, cleaning agent, preservatives, fire retardants, fertilizers, insecticides, and herbicides [15]. Boron can improve the antioxidant defense in vivo with its interesting biological function [17, 18]. When BA is administered at low doses, it protects cell membranes by enhancing antioxidant activity [19]. Nielsen et al. reported that the BA led to an increased resistance of DNA to oxidative damage induced by Aflatoxin B1 [20]. BA was found to strongly reduce the genotoxic effects of the lead and cadmium metals in V79 cells [21].

In a study investigating the protective effect of BA in ethanol-induced kidney injury, BA showed the damage by reducing the levels of MDA, TOS, and OSI. In addition, caspase-3 and TUNEL activities were decreased compared to the control group [19]. In another study, the protective effect of the application of BA in post-ischemic reperfusion injury of renal tissue was evaluated using various parameters on oxidative stress, renal inflammation, and apoptosis. Consequently, increased oxidative stress, inflammation, and apoptosis decreased after I/R treatment with various doses of BA. The antiapoptotic, anti-inflammatory, and antioxidant effects of the high-dose BA application were lower than those of the low-dose groups [22].

The potentially beneficial effects of various compounds are being studied in detail to prevent or mitigate the toxic effects caused by OTA. OTA is a ubiquitous nephrotoxic compound. It occurs as a mycotoxin in food and feed throughout the world. A large number of previous studies have shown that the most important characteristic of OTA is its nephrotoxicity in experimental animals. In morphological terms, OTA-induced renal damage in animals is characterized by atrophy of the proximal tubules and sclerosis of the interstitial cortex. OTA is thought to be involved in the etiology of Balkan endemic nephropathy—a human kidney disease related to specific areas—and in the etiology of urothelial tumors, which are very frequent in these areas [23–25]. For this reason, we selected the healthy HEK293 cell line in our studies to most closely mirror the potential toxicity of OTA. The aim of the present study is to evaluate the protective effect of BA on OTA-induced human embryonal kidney cells.

## Material and Methods

### Experimental Design

BA (CAS No. 10043–35-3, Sigma-Aldrich® Chem. Co. St. Louis, MO, USA) that was prepared with distilled water tested concentration (0.5, 5, and 50 μM) was selected according to the previous studies [20, 26]. OTA (C_20_H_18_ClNO_6_, CAS 303–47-9, Sigma-Aldrich® Chem. Co. St. Louis, MO, USA) was dissolved in a mixture of 95% ethanol and was added at concentrations of 25 μM in cell cultures [27, 28]. This concentration of OTA was selected according to previous studies that demonstrated cytotoxic and genotoxic effects in different cell lines exposed to OTA [29, 30]. However, Mitomycin C (CAS number 50–07-7; 10^−7^ M) was used as the positive control in the cytotoxic and genotoxic assay. Hydrogen peroxide (CAS number 7722–84-1; H_2_O_2_; 25 μM) and ascorbic acid (CAS number 50–81-7; 10 μM) were also used as the positive controls in oxidant and antioxidant analysis, respectively. The compounds for determining biochemical analysis and genotoxic effects were incorporated into the blood cultures following methods as mentioned below.

### Cell Culture and Cytotoxicity Assay

HEK293 cells were cultivated for 48 h at 37 °C in 96-well microplates with an initial concentration of 1 × 10^5^ cells/mL. Then, cells were treated with OTA (25 μM), BA (0.5, 5, and 50 μM), and their combinations incubated at 37 °C in a humidified 5% CO_2_ for 24 h. MTT [3-(4,5-dimethyl-2-thiazolyl)-2,5-diphenyl-2H-tetrazolium bromide] was added to the cell cultures for 3 h according to the manufacturer’s instructions (Cayman Chemical Company, USA), and the plates were measured at 570 nm using an ELISA plate reader (BMG Labtech, Ortenberg, Germany) [31].

### Lactate Dehydrogenase (LDH) Cytotoxicity Assay

LDH is a cytosolic enzyme that is a sign of toxicity caused by the external or internal factor in cells. Afterward, LDH reduces nicotinamide adenine dinucleotide (NAD^+^) to nicotinamide adenine dinucleotide reduced (NADH) and HS via the oxidation of lactate to pyruvate. LDH leakage assay was determined using the LDH Assay Kit (Cat no. ab102526, Abcam, Cambridge, UK) on the culture medium of a new set of cells exposed to OTA (25 μM), BA (0.5, 5, and 50 μM), and their combinations for 24 h. One hundred microliter of culture medium was transferred to a new 96-well plate. One hundred microliter of LDH reaction solution to each well was added, and absorbance was measured at 490 nm using an ELISA plate reader (BMG Labtech, Ortenberg, Germany) after 30 min [31].

### Total Antioxidant Capacity (TAC) and Total Oxidative Stress (TOS) Activity

TAC and TOS levels were measured in cellular media using a commercial kit (Rel Assay Diagnostics®, Gaziantep, Turkey) according to the manufacturer’s instructions [31]. Another group of cells for these experiments was treated with OTA (25 μM), BA (0.5, 5, and 50 μM), and their combinations and incubated at 37 °C in a humidified 5% CO_2_ for 2 h.

In TAC assay, potential antioxidants in culture medium cause a reduction of ABTS (2,2′-azino-bis 3-ethyl benzothiazoline-6-sulfuric acid) radical. Briefly, 500 μL of Reagent 1 solution in the kit content was added to the quartz cuvette containing 30 μL of plasma sample, and the initial absorbance was measured at 660 nm after 30 s. Then, 75 μL of Reagent 2 solution was added to the same cuvette and the absorbance was measured at 660 nm after 5 min incubation. The assay was calibrated with Trolox, and the results were expressed in terms of mM Trolox equivalent per liter (mmol Trolox Equiv/L).

TOS assay was based on the conversion of ferrous ion–chelator complex to ferric ion via oxidants present in the culture medium. To determine the TOS level, 500 μL of Reagent 1 was mixed with 75 μL of each plasma sample and the absorbance of each sample was measured at 530 nm after 30 s. Then, 15 μL of Reagent 2 was added to the mixture and the absorbance was again read at 530 nm. The treatments were calibrated with H_2_O_2_ and data were expressed as μM H_2_O_2_ equivalent per liter (μmol H_2_O_2_ Equiv/L).

### Micronucleus (MN) Assay

MN assay was carried out under procedures previously described by Robbiano et al. [32]. Five hundred microliter of the blood sample and OTA (25 μM), BA (0.5, 5 and 50 μM), and their combinations were added to 7 mL of Chromosome Medium B (Biochrom, Leonorenstr. 2–6.D-12247, Berlin) containing 100 U/mL penicillin, 100 µg/mL streptomycin, and 0.5 mL of phytohemagglutinin (Biochrom) and cell culture was incubated at 37 °C for 72 h. Cytochalasin B (Sigma) was added to the culture medium at 44 h of incubation. After the incubation period, the culture medium was centrifuged at 900 × g for 10 min and lymphocyte cells obtained were hypotonized by 0.075 M of cold potassium chloride for 30 min and then cells were fixed with ice‐cold methanol/acetic acid (3:1, v/v). The fixed cells were placed directly on slides using a cytospin and stained with Giemsa solution. The count of MN cells was performed under a light microscope by criteria declared by Robbiano et al. [32]. At least 2000 binucleated cells were counted per concentration (duplicate cultures for each concentration) for the formation of one, two, or more MN.

### Chromosome Aberration (CA) Method

The CA method for lymphocyte culture was performed with slight modifications of the previous procedure [33]. The blood sample (0.5 mL) and the concentrations described above of DMAA and cDMAA were cultured with 6 mL of Chromosome Medium B (Biochrom, Berlin) for 72 h at 37 °C. Two hours before the end of the incubation period, colchemide solution (0.1 mL) was added to the culture. After the incubation period, cells were collected by centrifugation and treated with a hypotonic solution (0.075 M KCl). Cells were re-incubated and centrifuged. A fixation solution (methanol to acetic acid, 3:1 v/v) was added to the cell suspension, and the resulting cells were resuspended and dropped onto clean slides. To prepare slides, a few drops of the fixed cell suspension were dropped onto the cold slide and air-dried. The slides were stained with Giemsa stain in phosphate buffer (pH 6.8) and allowed to dry. The evaluation process was performed by counting the fifty-metaphase plate showing different chromosome anomaly.

### Cell Cycle Analysis

HEK293 cells were seeded in 6-well plates at 1 × 10^4^ cells/mL for 48 h and treated various concentrations (0.5, 5, and 50 μM) of BA, OTA, and their combinations. The cell cycle phase was realized using a Muse™ Cell Cycle Assay Kit (Merck Millipore, Germany) according to the manufacturer’s instructions [34]. Briefly, cells were trypsinized with PBS and fixed by 70% cold ethanol. Muse cell cycle reagent was added to the obtained cell pellet and incubated for 30 min. The G0/G1, S, and G2/M percentage of cells was calculated by the Muse cell cycle analyzer (Merck Millipore, Germany).

### cDNA Synthesis and Quantitative Real-Time PCR Analysis

The effect of various concentrations (0.5, 5, and 50 μM) of BA, OTA, and their combinations on expression of Bax, Bıd, Bcl2, caspase-3, caspase-8, caspase-9, TNF, FAS, TP53, DFFA, NfKB1, TNRSFS1A, and VGEF was determined by RT-qPCR analysis [35]. Briefly, A549 cells exposed to the IC50 value of compounds 3c-f, cisplatin, and their combinations for 48 h were harvested and total RNA was isolated using the TriPure isolation reagent (Roche, Basel, Switzerland, Cat. no. 11 667 157 001). The quality of the isolated RNA was controlled by NanoDrop (NanoDrop ND-2000c, Thermo Scientific, Waltham, MA, USA). First-strand cDNA was synthesized from total RNA with the Transcriptor First Strand cDNA Synthesis Kit (Roche, Cat. no. 04 379 012 001). Real-time polymerase chain reaction (RT-PCR) analysis was conducted on the Light Cycler v.1.5 instrument (Roche Applied Science) and performed with SYBR Green PCR Master Mix (Qiagen). The real-time PCR mixture contained 5 μL SYBR Green PCR Master Mix, 0.5 μL cDNA, and 0.3 μM primer pairs in a total volume of 10 µL (Table 1). Cycling conditions for the PCR reaction were as follows: initially 10 min at 95 °C, followed by 40 cycles of cyclic denaturation at 95 °C for 15 s, annealing at 59 °C for 1 min, and extension 13 s at 72 °C. The beta-actin was used as an endogenous control. Relative ratios were calculated by normalizing gene expression levels of each sample, and the experiment was performed with three duplicates. Results were calculated by using Ct method (2^−∆∆Ct^ method) [36].

**Table 1:** Specific genes of primers

Gene	Primers
BAX	F-TCAGGATGCGTCCACCAAGAAG
	R-TGTGTCCACGGCGGCAATCATC
BID	F-TGGGACACTGTGAACCAGGAGT
	R-GAGGAAGCCAAACACCAGTAGG
BCL2	F-ATCGCCCTGTGGATGACTGAGT
	R-GCCAGGAGAAATCAAACAGAGGC
Caspase-3	F-GGAAGCGAATCAATGGACTCTGG
	R-GCATCGACATCTGTACCAGACC
Caspase-8	F-AGAAGAGGGTCATCCTGGGAGA
	R-TCAGGACTTCCTTCAAGGCTGC
Caspase-9	F-GTTTGAGGACCTTCGACCAGCT
	R-CAACGTACCAGGAGCCACTCTT
TNF	F-CTCTTCTGCCTGCTGCACTTTG
	R-ATGGGCTACAGGCTTGTCACTC
FAS	F-GGACCCAGAATACCAAGTGCAG
	R-GTTGCTGGTGAGTGTGCATTCC
TP53	F-CCTCAGCATCTTATCCGAGTGG
	R-TGGATGGTGGTACAGTCAGAGC
DFFA	F-TCAGACCTTGGGAGACAACACG
	R-CGAAGGTGACTCTCGCTATTCC
NfKB1	F-GCAGCACTACTTCTTGACCACC
	R-TCTGCTCCTGAGCATTGACGTC
TNFRS1A	F-CCGCTTCAGAAAACCACCTCAG
	R-ATGCCGGTACTGGTTCTTCCTG
VGEF	F-TTGCCTTGCTGCTCTACCTCCA
	R-GATGGCAGTAGCTGCGCTGATA

### HET-CAM (Hen’s Egg Test Chorioallantoic Membrane) Irritation Test

The irritant effect of algae extracts was demonstrated using a chorioallantoic membrane model on fertilized hen’s eggs. The irritant effects of the samples were tested on fertilized Leghorn chicken eggs weighing 50–60 g from commercial sources (Lezita, İzmir, Turkey) using the HET-CAM method modified according to Kishore et al. [37] and Güner and Karabay Yavasoglu [38]. The fertilized chicken eggs were placed in an incubator with a conveyor belt rotation system at 37 ± 1 °C and 80 ± 2% humidity for 7 days. On day 7, the eggs were opened on the stump side and aspirated through a hole on the pointed side. A round piece of the shell (3–4 cm in diameter) was then carefully removed with forceps. The inner membrane was then carefully removed with forceps without damaging the blood vessels. Then, 300 mL of the freshly prepared sample at a concentration of 0.5 and 1 mg/mL dissolved in DMSO (0.05%) (0.5 to 1 mg/mL) was applied to the CAM. The severity of irritation (IS) for a period of up to 5 min was assessed as follows:

$${\text{IS}}=\left[\left(301-h\right)\times 5\right]/300+[(301-h)\times 7]/300+[(301-c)\times 9]/300$$

where *h* is the time vascular hemorrhage occurred, *l* is the time the first vascular lysis occurred, and *c* is the time the first vascular coagulation occurred. Irritation classification is based on IS: 0.0–0.9, non-irritation; 1.0–4.9, slight irritation; 5.0–8.9, moderate irritation; 9.0–21.0, severe irritation. In addition, 0.9% NaCl was tested as a negative control and 0.1 N NaOH was tested as a positive control at a concentration of 300 µL. For each test compound five eggs were used. All samples were tested in triplicate at different time points.

### Statistical Analysis

Statistical analysis was performed using SPSS 20.0 (SPSS, Chicago, IL, USA). The experimental data were analyzed by one-way analysis of variance (ANOVA), and Duncan’s test was performed to examine whether there were any differences between the application and control groups. Pearson’s *r* coefficient was used to determine correlations between data. The results are presented as means ± SD of at least three independent experiments, and *p* < 0.05 was accepted as significant. All assays were run in triplicate.

## Results

### Cell Viability and LDH Activity

**Fig. 1:**
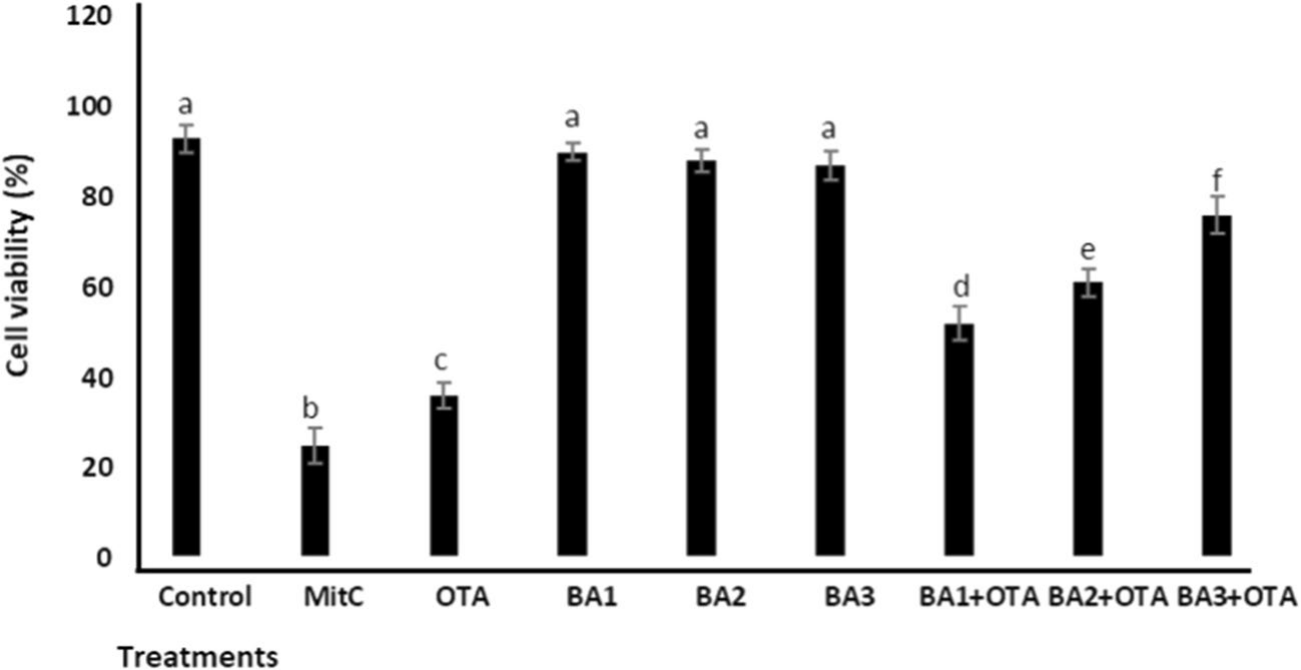
Cell viability rates of HEK293 cells that are treated with OTA (25 μM) and boric acid (0.5, 5, and 50 mg/L) alone or in the presence of OTA. Values represent means ± SD of at least three experiments. Bars indicated by the different letters show significant differences at the *p* < 0.05 level. OTA: ochratoxin A, BA1: 0.5 μM dose of boric acid, BA2: 5 μM dose of boric acid, BA3: 50 μM dose of boric acid

The viability of lymphocytes exposed to OTA and BA was determined by MTT assay (Fig. 1). The result showed that cell viability did not change after treatment of BA, while significantly (*p* < 0.05) inhibiting after treatment of OTA (2.58-fold) compared to the untreated control. Concentrations of 0.5, 5, and 50 μM of BA significantly increased (*p* < 0.05) cell viability with a 1.44-, 1.69-, and 2.11-fold changes compared to treatment with OTA alone, respectively.

**Fig. 2:**
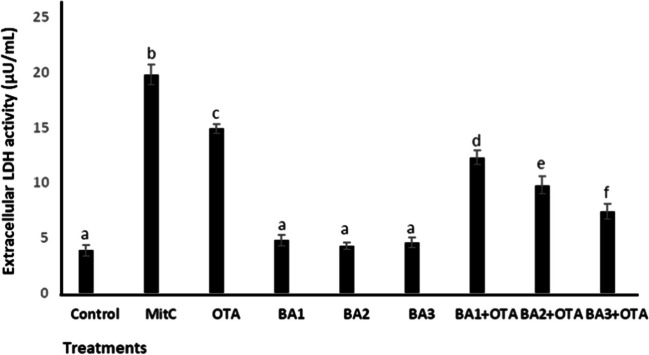
Extracellular LDH activity of HEK293 cells that are treated with OTA (25 μM) and boric acid (0.5, 5, and 50 mg/L) alone or in the presence of OTA. Mitc (10^−7^ M) was used as positive control. Values represent means ± SD of at least three experiments. Bars indicated by the different letters show significant differences at the *p* < 0.05 level. OTA: ochratoxin A, BA1: 0.5 μM dose of boric acid, BA2: 5 μM dose of boric acid, BA3: 50 μM dose of boric acid

To verify the cytotoxic effects, the damage in the cell membrane after treatment was assessed by LDH assay. As shown in Fig. 2, OTA caused a significant increase (*p* < 0.05) in LDH levels with a value of 3.82 compared to the untreated control, while BA resulted in no change (*p* > 0.05). Combination treatment of BA at 0.5, 5, and 50 μM concentrations with OTA, LDH levels decreased significantly (*p* < 0.05) with a value of 1.21, 1.52, and 2.01 compared to treatment with OTA alone, respectively.

### TAC and TOS Activity

**Fig. 3:**
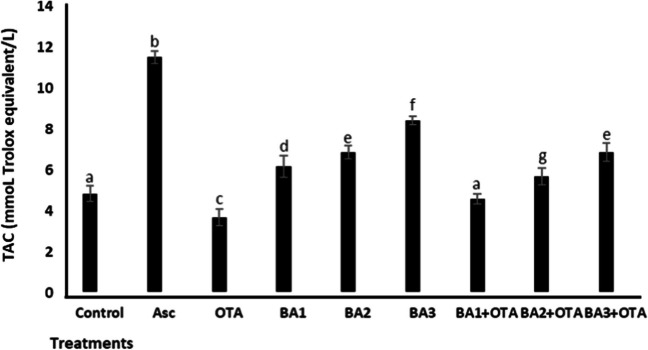
Total antioxidant capacity of HEK293 cells that are treated with OTA (25 μM) and boric acid (0.5, 5, and 50 mg/L) alone or in the presence of OTA. H_2_O_2_ (25 μmol/L) and ascorbic acid (10 μmol/L) were used as positive controls for TAC activity. Values represent means ± SD of at least three experiments. Bars indicated by the different letters show significant differences at the *p* < 0.05 level. OTA: ochratoxin A, BA1: 0.5 μM dose of boric acid, BA2: 5 μM dose of boric acid, BA3: 50 μM dose of boric acid

**Fig. 4:**
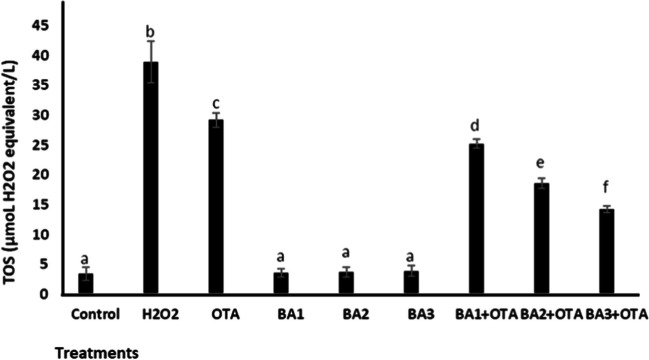
Total oxidative stress levels in HEK293 cells that are treated with OTA (25 μM) and boric acid (0.5, 5, and 50 mg/L) alone or in the presence of OTA. H_2_O_2_ (25 μmol/L) and ascorbic acid (10 μmol/L) were used as positive controls for TOS activity. Values represent means ± SD of at least three experiments. Bars indicated by the different letters show significant differences at the *p* < 0.05 level. OTA: ochratoxin A, BA1: 0.5 μM dose of boric acid, BA2: 5 μM dose of boric acid, BA3: 50 μM dose of boric acid

The changes in the total amount of antioxidants and oxidants in renal cells after exposure to OTA and BA were analyzed by the TAC and TOS assays. As shown in Fig. 3 and Fig. 4, OTA caused a significant decrease (*p* < 0.05) in TAC levels (1.31-fold decrease) and an increase in TOS (8.53-fold increase) compared to the untreated control, respectively. BA caused a significant increase (*p* < 0.05) in TAC levels by 1.27-, 1.41-, and 1.73-fold, respectively, compared to the untreated control, while TOS levels did not change (*p* > 0.05). When analyzing BA treatments with concentrations of 0.5, 5, and 50 μM, TAC values increased significantly (*p* < 0.05) by 1.25-, 1.54-, and 1.86-fold, respectively, and TOS values decreased by 1.15-, 1.57-, and 2.05-fold, respectively, compared to treatments with OTA alone.

### MN and CA Assay

**Fig. 5:**
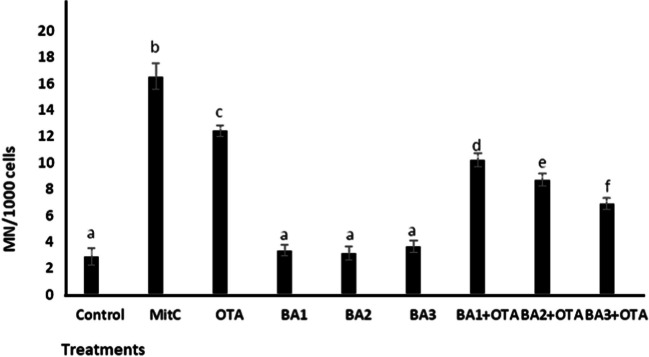
Micronucleus (MN) assay in HEK293 cells that are treated with OTA (25 μM) and boric acid (0.5, 5, and 50 mg/L) alone or in the presence of OTA. Values represent means ± SD of at least three experiments. Bars indicated by the different letters show significant differences at the *p* < 0.05 level. OTA: ochratoxin A, BA1: 0.5 μM dose of boric acid, BA2: 5 μM dose of boric acid, BA3: 50 μM dose of boric acid

**Fig. 6:**
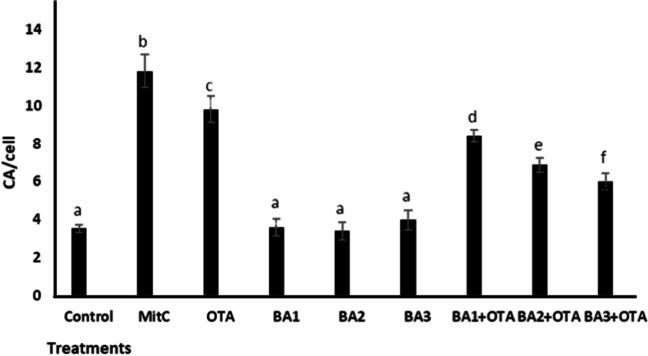
Chromosome aberration of HEK293 cells that are treated with OTA (25 μM) and boric acid (0.5, 5, and 50 mg/L) alone or in the presence of OTA. Mitc (10^−7^ M) was used as positive control. Values represent means ± SD of at least three experiments. Bars indicated by the different letters show significant differences at the *p* < 0.05 level. OTA: ochratoxin A, BA1: 0.5 μM dose of boric acid, BA2: 5 μM dose of boric acid, BA3: 50 μM dose of boric acid

As shown in Fig. 5 and Fig. 6, genotoxic effects were detected in  HEK293 cells after exposure to OTA and BA by MN and CA methods. OTA caused a significant increase (*p* < 0.05) in the frequency of MN (4.7-fold increase) and CA (2.76-fold increase) compared to the untreated control. BA did not change (*p* > 0.05) the frequency of MN and AC. When analyzing BA treatments with concentrations of 0.5, 5, and 50 μM, MN frequency decreased significantly (*p* < 0.05) by 1.21-, 1.42-, and 1.73-fold and CA frequency by 1.6-, 1.42-, and 2.04-fold, compared to treatments with OTA alone (Fig. 5 and Fig. 6).

### Cell Cycles

**Fig. 7:**
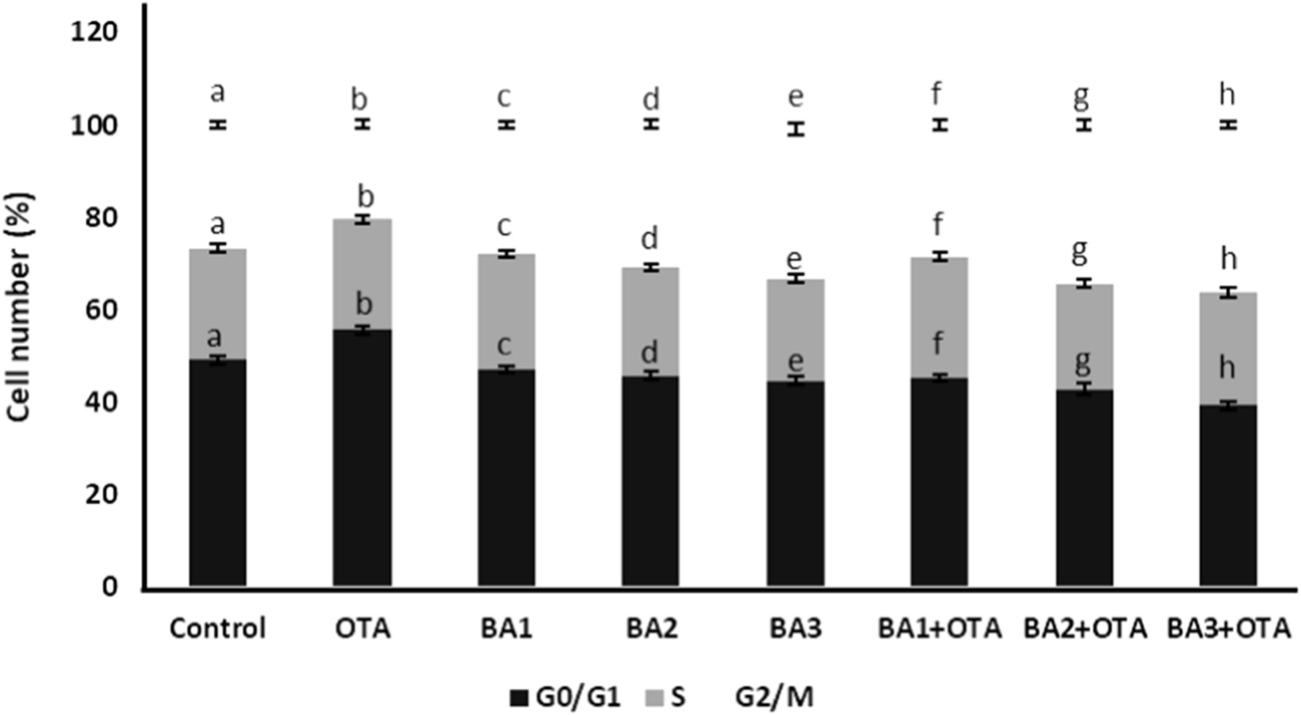
Determination of cell number during cell cycles G0/G1, S, and G2 in HEK293 cells that are treated with OTA (25 μM) and boric acid (0.5, 5, and 50 mg/L) alone or in the presence of OTA. Values represent means ± SD of at least three experiments. Bars indicated by the different letters show significant differences at the *p* < 0.05 level. OTA: ochratoxin A, BA1: 0.5 μM dose of boric acid, BA2: 5 μM dose of boric acid, BA3: 50 μM dose of boric acid

OTA alone significantly (*p* < 0.05) decreased cell population in the G2/M cycle with a 1.3-fold change. BA at concentrations of 0.5, 5, and 50 μM significantly increased (*p* < 0.05) with 1.04, 1.16, and 1.2-fold changes in G2/M population compared to control, followed by a decrease in cell population in the G0/G1 phase (Fig. 7). Combination treatments of 0.5, 5, and 50 μM BA concentration with OTA significantly increased the cell population in the G2/M cycle (*p* < 0.05) with a value of 1.39, 1.68, and 1.78-fold changes, respectively, compared with OTA alone (Fig. 7).

### Gene Expression Profile

As can be seen in Table 2 in relation to the analysis of gene expression associated with BA treatment in an OTA-induced kidney cell line, OTA upregulated the VGEF, BCL2, NF-κB1 antiapoptotic gene expressions (with a fold 1.1 to 5.2). Proapoptotic gene expressions such as TNF, FAS, TNFRSF1A, BAX, BID, Casp-3,-8,-9, and TP53 genes (with a fold change value of 1.3, 1.3, 2.1, 1.2, 2.9, 1.2, 3.4, and 1.1, respectively) were upregulated by combination with OTA of BA in a dose-dependent manner.

**Table 2:** Gene expression profile associated with BA treatment in an OTA-induced kidney cell line

Gene symbol	OTA	BA1	BA2	BA3	OTA + BA1	OTA + BA2	OTA + BA3
TNF receptor							
TNF	− 1.4	1.3	1.8	2.4	− 1.1	1.5	2.1
FAS	− 2	1.6	1.3	1.4	1.3	1.2	1.6
VGEF	2.01	2.4	− 3.8	− 2.6	1.3	− 4.6	− 2.1
TNFRSF1A	− 5.6	2.1	2.4	2.2	1.3	7.3	6.2
BCL2 family antiapoptotic members							
BCL2	5.2	− 1.1	− 3.1	− 2.4	− 1.6	− 1.8	− 2.3
Pro-apoptotic members							
BAX	1.2	1.6	1.4	1.9	2.1	3.6	6.2
BID	− 1.3	− 4.1	− 2.3	− 1.1	1.2	1.2	1.3
NF-kB family							
NFkB1	2.1	− 1.2	− 1.1	− 1.8	− 1.4	− 1.1	− 2.1
Caspases and regulators							
CASP3	− 1.2	1.1	1.4	1.3	2.9	1.2	3.7
CASP8	− 2.3	2.1	1.7	7.8	1.2	3.3	2.5
CASP9	− 3.2	1.2	2.7	1.6	3.4	2.1	7.8
Other							
DFFA	− 5.6	− 2.1	− 2.5	1.1	− 1.1	− 1.1	− 2.1
TP53	− 2.1	1.2	− 1.1	1.3	1.1	1.6	1.7

### HET-CAM Irritation Test

**Fig. 8:**
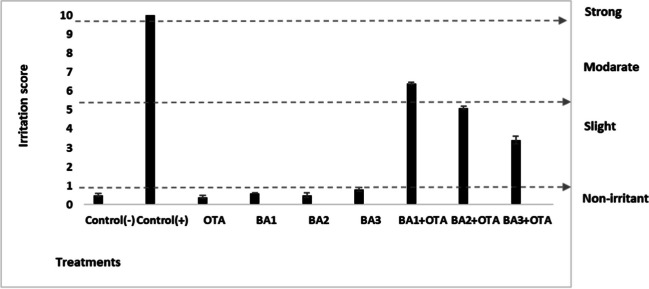
Irritation scores after treatments of OTA (25 μM) and boric acid (0.5, 5, and 50 mg/L) and their combinations on membrane surface up to 5 min. Values represent means ± SD of at least three experiments. Bars indicated by the different letters show significant differences at the *p* < 0.05 level. OTA: ochratoxin A, BA1: 0.5 μM dose of boric acid, BA2: 5 μM dose of boric acid, BA3: 50 μM dose of boric acid

The effects of irritation on vascularization after exposure to OTA and BA are shown in Fig. 8. OTA caused hemorrhage and lysis and severe irritation with an IS score of 8.7 ± 1.8 for a period of up to 5 min. BA had no irritant effect at any concentration. Similarly, combined treatment of OTA with concentrations of 0.5, 5, and 50 μM of BA caused mild irritation with the IS score of 6.45 ± 1.5, 5.1 ± 1.3, and 3.4 ± 0.8, respectively.

## Discussion

OTA is a highly toxic mycotoxin and poses a significant risk to human health. Its severe toxicity and widespread contamination in foods of both plant and animal origin, such as cereals, wine, coffee, beer, cocoa, dried fruits, spices, meat, and milk, have led to considerable efforts to assess human exposure to OTA [39]. The relationship between oxidative stress and inflammation is well established in the literature, and oxidative stress is known to play a pathogenic role in chronic inflammatory diseases (Fig. 4). Oxidative stress increases the levels of proinflammatory cytokines and the expression of inflammatory molecules such as vascular cell adhesion molecule-1 (VCAM-1), intercellular adhesion molecule-1 (ICAM-1), and nuclear factor kappa B (NF-κB) in many pathogenic diseases, including OTA intoxication [40]. At higher doses, OTA also causes immunotoxicity, neurotoxicity, and teratogenicity. It accumulates in the kidneys and induces kidney and liver tumors in rodents when exposed to chronic renal toxic doses [41]. Several studies have demonstrated the chronic adverse effects of OTA in mammals even at low concentrations [42–44]. Repeated exposure to OTA impairs renal function and morphology and increases the incidence of renal adenomas and carcinomas due to increased oxidative stress induced by OTA. Cell viability assays showed significant cytotoxicity of OTA (25 μM) in HEK293 cell lines (Fig. 1). Boron has been shown to have antioxidant, anti-inflammatory, anticancer [45], hepatoprotective [46], antigenotoxic [47], cytotoxic, and apoptotic effects [48]. It limits oxidative damage by replenishing the body’s glutathione stores and inhibiting other reactive oxygen species [49]. In another study, boron directly inhibited LPO and increased GSH levels against oxidative stress induced by arsenic [50]. This study was driven by the idea of investigating the potential protective effect of BA against OTA-induced injury. We investigated the protective effect of BA on HEK293 cell lines using the MTT assay. In the cell viability assay, we confirmed the ability of BA to reduce OTA-induced cell death in a dose-dependent manner.

The toxic effect of mycotoxins can lead to oxidative stress (OS) and the formation of free radicals [51]. Biological free radicals are highly unstable molecules that react with various organic substrates such as lipids, proteins, and DNA [52]. In small to moderate amounts, ROS are useful for regulating processes to maintain homeostasis and for a variety of cellular functions. Excessive production of ROS leads to structural changes in cellular proteins and alteration of their functions, resulting in cellular dysfunction and disruption of vital cellular processes. High levels of ROS cause lipid, protein, and DNA damage. In particular, ROS can disrupt the lipid membrane and increase membrane fluidity and permeability [53]. The loss of cell viability after OTA exposure could be due to the impairment of cell membrane integrity caused by high ROS production leading to LDH leakage. To confirm this hypothesis, we examined TAC, TOC, and LDH levels after OTA treatment. Our results showed that ROS and LDH were increased and TOC decreased in renal cells treated with OTA, while treatment of renal cells with BA limited the release of LDH into the culture medium and TOS levels. In addition, BA led to an increase in TAC levels. The antioxidant capacity of BA, which is a known component of cell membrane functions and enzymatic reactions, has not yet been fully elucidated to date [54, 55]. One study showed that daily boron supplementation of 100 mg/kg reduced lipid peroxidation by increasing antioxidant activity [56]. Similarly, Ince et al. reported that a high amount (10.8 mg/kg) of additional BA in the diet probably has no adverse effects on intestinal motility [57].

The battery of tests to evaluate the genotoxicity of OTA gave negative results, but some positive results were found in some in vitro and in vivo studies, such as DNA breaks in mammalian cell lines, DNA damage and micronuclei in primary cultures of human and rat kidney cells, and cytogenetic damage and DNA adducts in rats treated with OTA [58]. As expected, OTA led to an increase in MN and CA abundance in HEK293 cells. More importantly, BA provided effective protection against OTA-induced genotoxicity. There are similar findings in the literature that BA can reduce toxin-induced genotoxicity [20, 21, 59]. Biologically important macromolecules in mammalian cells, such as nucleic acids and proteins, are protected by antioxidants [60]. Our results show that BA has antigenotoxic and antioxidant properties at concentrations of 0.5 to 50 μM (Fig. 3).

There exist three major cell-cycle checkpoints: the G1/S checkpoint, the G2/M checkpoint, and the spindle assembly checkpoint (SAC) (Fig. 7). G2/M arrest is a phase of the cell cycle in which cells are temporarily arrested in G2 before entering mitosis. This arrest is important for several reasons. First, it allows cells to ensure that DNA replication and repair processes are properly completed before entering mitosis, preventing the transfer of damaged DNA to daughter cells. Second, G2/M arrest allows cells to monitor the integrity of the genome and activate DNA damage response pathways when needed. Finally, G2/M arrest is critical for proper cell division and maintenance of genomic stability. Agents that induce G2/M arrest may be beneficial in the treatment of cancer by preventing the proliferation of cancer cells with DNA damage, leading to cell death or sensitizing them to other therapies [61, 62]. In our study, there was a decrease in the cell population in the G2/M phase after OTA application. The application of BA induced a G2/M arrest in the cell cycle in a dose-dependent manner.

The potential irritability of compounds was assessed using the HE-CAM method (Fig. 8). The test method is based on the observation that the CAM of an embryonated chicken egg is similar to the vascularized mucosal tissues of the eye [37]. OTA caused an irritant effect after hemorrhagic and lytic damage in the vascular system. Some studies have shown that mycotoxins can cause cerebral vascular damage and cardiovascular dysfunction [63]. However, BA mitigates the irritant effects caused by OTA. BA may have a vasoprotective effect with its cytoprotective and antioxidant properties. In one study, BA was found to help protect against myocardial infarction in mice [64].

OTA is considered a nephrotoxin for animals and humans and is a very potent liver toxin, strong teratogen, carcinogen, and immunosuppressant [41]. OTA causes mitochondrial damage, oxidative stress, lipid peroxidation, and apoptosis in cells and model animals such as rats, mice, and pigs when chronically ingested [65–67]. Apoptosis normally occurs during development and aging and as a homeostatic mechanism to maintain cell populations in tissues. Apoptosis also occurs as a defense mechanism, e.g., in immune responses or when cells are damaged by disease or pollutants [68]. Over the past decade, naturally occurring dietary agents known to have chemopreventive effects in experimental models have been shown to target signaling mediators in apoptosis-triggering metabolic pathways. Apoptosis is triggered by two different signals, an extrinsic one, which mainly responds to extracellular stimuli, and an intrinsic one, which is activated by modulators within the cell itself. Proapoptotic agents could protect against cancer by enhancing the elimination of initiated precancerous cells, and antiapoptotic agents could promote tumor formation by inhibiting apoptosis in genetically damaged cells [69]. The present study is the first to reveal the mechanisms of anticancer/antiproliferative effect of BA via the different apoptotic pathways in the OTA-induced HEK293 cell line. In this context, BA treatments contributed to the regulation of 59 apoptotic genes related to the tumor suppressor p53 (TP 53), the extrinsic/intrinsic receptor pathway, and the transcription factor NfKB1 pathway. In our study, gene expression profiles were used to compare the characteristics of the differentially expressed genes. After treatment of HEK293 cells with OTA, the genes TNF, FAS, TNFRSF1A, BID, CASP-3,-8,-9, DFFA, and TP53 were downregulated and the genes VGEF, BCL-2, BAX, and NFkB1 were upregulated. In the study of OTA-induced HEK293 cells after BA treatments, the genes TNF, FAS, TNFRSF1A, BAX, BID, CASP-3,-8,-9, DFFA, and TP53 were upregulated and the genes VGEF, BCL-2, and NFkB1 were downregulated. The caspase cascade system plays a central role in the regulation of apoptosis, and changes in genes of this pathway are crucial for the fate of the extrinsic pathway. Caspases-3, 8, and 9 play a crucial role in controlling the traffic of the apoptosis signaling pathway. Caspase-8 can trigger the signaling of the caspase activation cascade via the extrinsic pathway [70]. In this study, treatment with BA resulted in upregulation of Casp-3,-8,-9, indicating persistent stimulation of the extrinsic pathway. In other words, the extracts tend to accelerate cancer cell apoptosis via the extrinsic pathway. The intrinsic pathway plays a crucial role in regulating apoptosis in response to cellular stress, and this pathway is controlled by the anti- and pro-apoptotic genes of the Bcl-2 families. Inappropriate overexpression of the apoptosis-suppressing Bcl-2 protein has been shown to contribute to tumorigenesis in various tumor types. The apoptosis-preventing effect of Bcl-2 is counteracted by the proapoptotic protein Bax. An imbalance in the Bax/Bcl-2 ratio, which tips the scales towards survival, can make tumor cells more resistant to a variety of cell death stimuli, including all chemotherapeutic agents, radiation, hypoxia, or growth factor deprivation [71]. The administration of BA led to a decrease in the expression of BCL2 and an increase in the expression of the BAX and BID genes. The DFFA subunit acts as a substrate for caspase-3, which cleaves and activates FFFA, leading to DNA fragmentation during apoptosis [72]. An increase in DFFA gene expression was observed after BA treatment. BA promoted apoptosis by increasing the expression of several genes of the intrinsic pathway, which is downstream of the extrinsic pathway. In addition, BA led to activation of the TP53 gene. One study showed that inhibition of the NF-kB signaling pathway induced apoptosis in synovial cells and suppressed cell proliferation and angiogenesis [73]. Similarly, in our study, a decrease in NFkB1 gene expression was observed after BA administration.

## Conclusion

Our results showed that boric acid treatment led to different gene expression on the same pathway. Upregulations in pro-apoptotic gene expressions such as TNF, TNFRSF1A, FAS, BAX, and Casp-3,-8,-9, and TP53 after boric acid treatment in a dose-dependent manner suggested that apoptosis is active through the intrinsic and extrinsic pathway. Moreover, downregulations in antiapoptotic gene expressions such as NfKB1 (in BA1 and BA2 doses), BCL2, and DFFA revealed that boric acid tend to induce apoptosis by blocking antiapoptotic gene following activating pro-apoptotic genes. However, downregulations in some proapoptotic gene expressions (BID gene and TP53 gene in BA2 dose) and upregulations in some antiapoptotic gene expressions (DFFA in BA3 dose) may be associated with the tendency of the escape from apoptosis of cells or the relevance for the survival of the cells or changes in the post-translational modifications. More detailed gene expression in intrinsic and extrinsic pathways is needed to elucidate this mechanism.

In conclusion, the results of this study demonstrate that BA was effective for the prevention of OTA-induced toxic effects in human embryonal kidney cells (HEK293). The results of this study show that the protective effects of BA may be caused both by an increase in the activity of the antioxidant defense system. Also, BA protects different gene expressions, induced to apoptosis.

## Data Availability

No datasets were generated or analyzed during the current study.
